# Supplementary material for: Energy scaling of targeted optimal control of complex networks
Source: Nat Commun. 2017 Apr 24;8:15145. doi: 10.1038/ncomms15145 (PMC5413984; doi:10.1038/ncomms15145)
Supplement: Supplementary Information — Supplementary Figures, Supplementary Tables, Supplementary Notes and Supplementary References. [file ncomms15145-s1.pdf]

Supplementary Figures

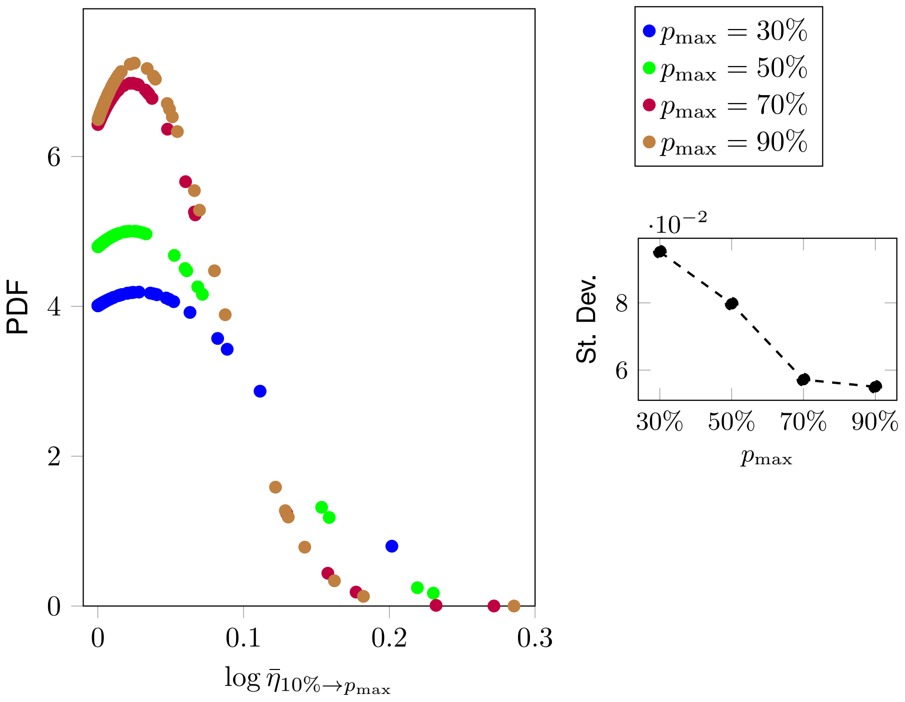

Supplementary Figure 1. Computing η for different values of p_{min} and p_{max}. From the Methods section we see that η may be computed from one target set size to another (which we call p_{min} and p_{max}). To ensure that we compute a value of η that describes the entire network, we keep $p_{\text{min}} = 10\%$ and compute values of $\log \bar{\eta}_{p_{\text{min}} \to p_{\text{max}}}$ for larger values of p_{max}. We see that the distributions as p_{max} increases becomes 'sharper', i.e., that the standard deviation decreases, which is shown in the inset plot. After p_{max} grows larger than 70%, we see that the improvement of the computed $\log \bar{\eta}_{p_{\text{min}} \to p_{\text{max}}}$ slows down so that we do not need to compute η_i for many additional points.

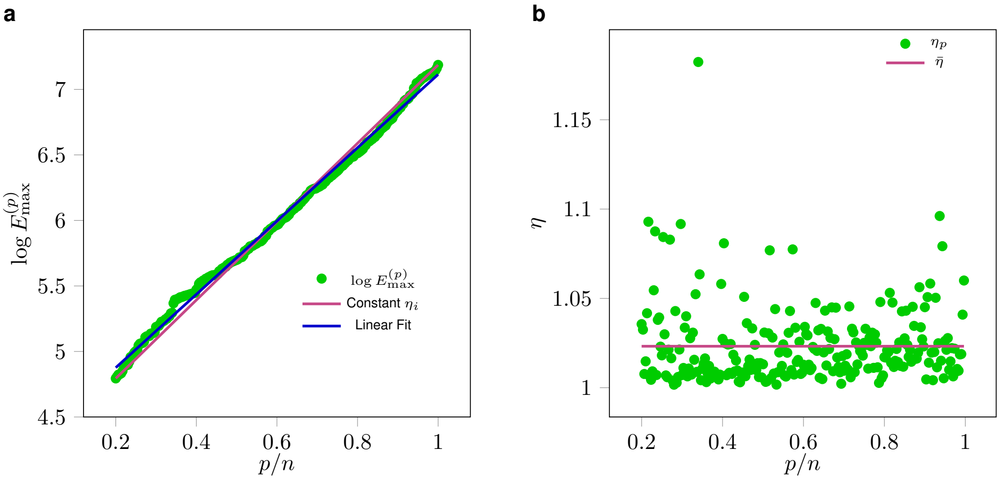

Supplementary Figure 2. The ratio of maximum energies is approximately constant. For a network, we compute each value of η iteratively as the cardinality of the target set is reduced from n to 1. In panel **a**, we plot the individual values of $\log E_{\max}^{(p)}$ as p is varied and compare the trend to a line with the slope of η if each value of η_i is assumed constant and a linear fit for the values of $\log E_{\max}^{(p)}$. We see good agreement between the two methods. In panel **b**, we plot the individual values of $\eta_i = E_{\max}^{(p+1)}/E_{\max}^{(p)}$. The deviation around the mean is fairly small.

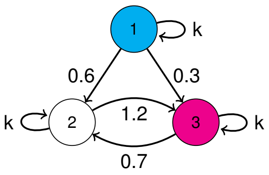

$$A = \begin{bmatrix} k & 0 & 0 \\ 0.6 & k & 0.7 \\ 0.3 & 1.2 & k \end{bmatrix}$$

$$B = \begin{bmatrix} 1 \\ 0 \\ 0 \end{bmatrix}$$

$$C = \begin{bmatrix} 0 & 0 & 1 \end{bmatrix}$$

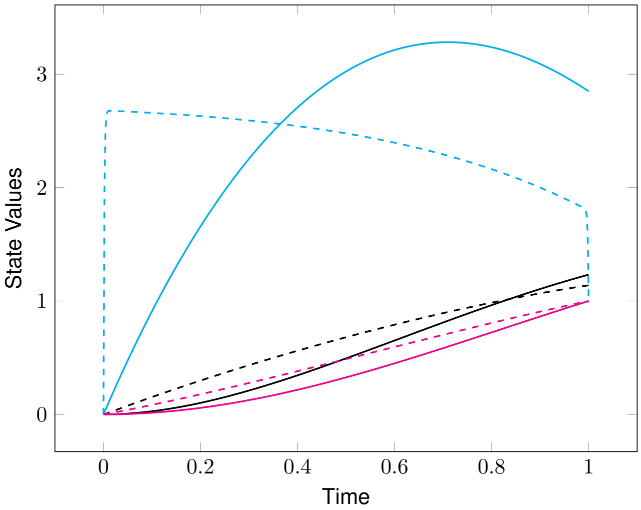

Supplementary Figure 3. An example of the uses of the state weight matrices. A three node network where node 1 is the driver, i.e., receives the control input, and node 3 is the target, i.e., the ouput of the system is the state of node 3, has an initial condition at the origin and a final condition when $y_f = x_3(t_f) = 1$. The solid lines correspond to minimum energy control, i.e., when $Q_1 = Q_2 = O_N$ and $\hat{R} = 1$. The dashed lines correspond to a cost function where a weight of 1000 is included for the derivatives $\dot{x}_2(t)$ and $\dot{x}_3(t)$ and a small weight is included for the control input, $\hat{R} = 0.001$. We can see that the rise of state one is more steep when a weight to the state derivative is included than for the minimum energy control trajectory. The state and control input weights can be tuned to achieve a desired state space trajectory.

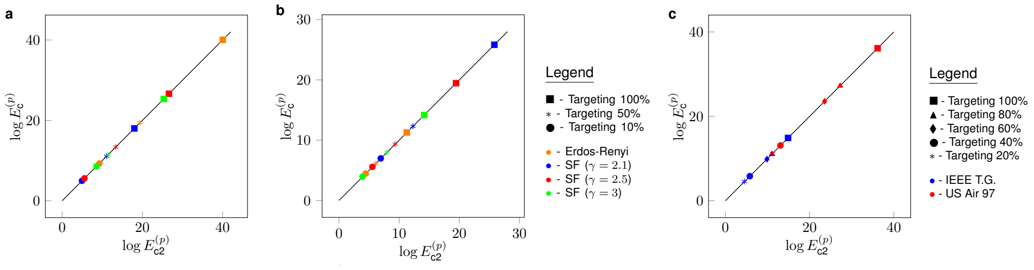

Supplementary Figure 4. Scaling of the total energy $E_{\mathbf{c}}^{(p)}$ versus the open loop energy $\tilde{\boldsymbol{\beta}}^T \tilde{W}_p^{-1} \tilde{\boldsymbol{\beta}}$. We show that for a variety of networks, real and model, scale-free and Erdos-Renyi, all nodes targeted or only some nodes targeted, the total energy for an arbitrary maneuver $\tilde{\boldsymbol{\beta}}$ is well approximated by the open loop energy. Note that this approximation holds best when the controllability Gramian is poorly conditioned. Each control input is calculated for a cost function where Q_1, Q_2 and R are appropriately dimensioned identity matrices. The model networks have the properties: $n = 100$, $\gamma_{\text{in}} = \gamma_{\text{out}} = 3.0$, $k_{\text{av}} = 5$, and $n_{\text{d}} = 0.5$. ***a*** *Low average degree,* $k_{av} = 2$. ***b*** *Moderate average degree* $k_{av} = 5$. The solid line has a slope of one. ***c*** *Two real networks.*

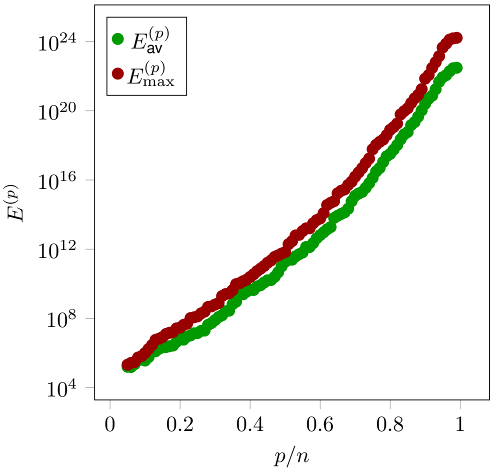

Supplementary Figure 5. Average Energy and Maximum Energy. The energy averaged over p control maneuvers $\boldsymbol{\beta}$ is shown in green for different values of p. The corresponding maximum energy is shown in red. Note that for any given p, the order of magnitude of the average energy is not much less than the order of magnitude of the maximum energy.

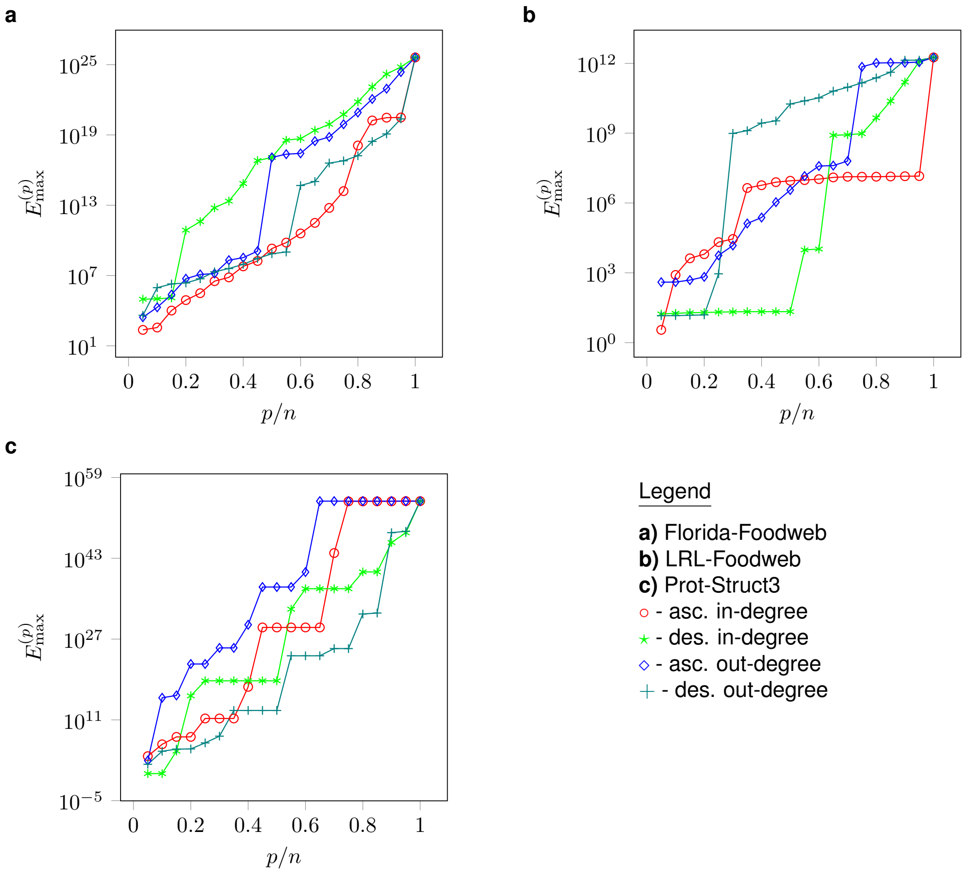

Supplementary Figure 6. Effects of different selections strategies for the target nodes. We plot $E_{\max}^{(p)}$ versus the target fraction p/n for three real networks: the Florida foodweb [1], the Little Rock Lakes region foodweb [2], and a protein structure [3]. Nodes were removed from the target set in four different ways: (i) ascending in-degree, (ii) descending in-degree, (iii) ascending out-degree, (iv) descending out-degree. For each network $n_{\rm d} = 0.45$.

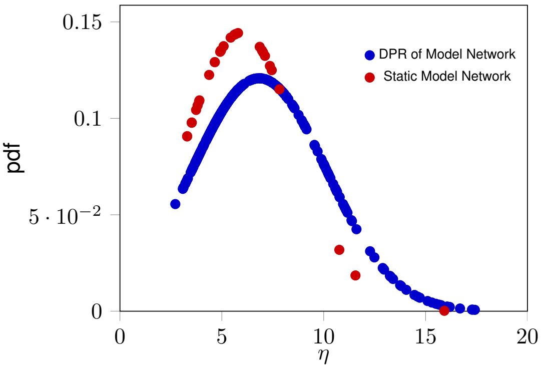

T-test statistics

	SM Network	DPR Network
mean	6.00	6.83
std. dev	2.76	3.30

significant level, $\alpha = 5\%$
p-value $= 0.1642$

Supplementary Figure 7. Model Network: T-test and p-value analysis: Probability density function (PDF) of the distributon of η of the model networks and their DPR versions. The T-test results are also presented.

Supplementary Tables

Real datasets from literature.

	Name	n	l	k_{av}	d	η
Circuit	s208st [3]	122	188	1.54	14	13.16
	s420st [3]	252	399	1.58	16	12.78
	s838st [3]	512	819	1.6	20	11.86
Citation	Kohonen [1]	3772	12731	3.38	9	6.32
	SG [1]	1024	4919	4.8	11	5.37
	SW [1]	233	994	4.27	7	5.84
	Scien [1]	2729	10413	3.82	13	6.12
Foodweb	Carpinteria [4]	128	2290	17.89	6	7.36
	Florida [1]	128	2106	16.45	5	5.14
	Grassland [1]	113	832	7.36	3	3.92
	LRL [2]	183	2494	13.63	6	4.29
	StMarks [1]	54	356	6.59	7	4.74
	Ythan [5]	92	417	4.53	3	5.77
Infrastructure	AirTrafficControl [6]	1226	2615	2.13	25	5.11
	IEEETG [7]	118	358	3.03	14	5.01
	NorthEuroGrid [8]	236	640	2.71	23	6.04
	USAir500 [9]	500	5960	11.92	9	4.29
Metabolic	CE_met [10]	1173	2864	2.44	30	13.24
	EN_met [10]	916	2176	2.38	28	14.72
	SC_met [10]	1511	3833	2.54	22	10.09
	TM_met [10]	830	1980	2.39	18	14.09
	TP_met [10]	485	1117	2.3	15	11.94
	Yu-11 (New) [11, 12]	1144	2293	2.0	16	50.83
	CCSB-YI1 (New) [12, 13]	1278	3450	2.7	14	24.06
ProtStruct	prot_struct_1 [3]	95	213	2.24	11	8.95
	prot_struct_2 [3]	53	123	2.32	6	7.0
	prot_struct_3 [3]	99	212	2.14	10	9.1
Social	EmailURV [14]	1133	10903	9.62	8	4.49
	FBForum [15]	899	7089	7.89	9	6.23
	Jazz [16]	198	5484	27.7	6	3.36
	RHS [17]	217	2672	12.31	6	3.67
	UCIrvine [18]	1899	20296	10.69	8	3.56

Supplementary Table 1. Both in the manuscript and here in the supplementary information, we examine how target control may benefit real networks compiled in datasets found throughout the scientific and engineering literature. We include the name, the reference, and some basic properties for each of the networks, as well as our computed value of η. In the table, n is the number of nodes, l is the number of edges, k_{av} is the average degree, d is the diameter of the graph, and η is the scaling of the minimum control energy as we discuss in the manuscript and in the supplementary information.

Supplementary Note 1. Introduction to supplement

Complex networks have recently been used to model many distributed systems such as food webs, communicating robots, financial interdependence, and social networks. While the dynamics of any one of those networks are rich in nonlinearities and uncertain parameters, we will restrict ourselves to linear dynamics. Linear dynamics are appropriate when a system is operating near a stable point, or if certain assumptions can be made. Also, the differences between the specific dynamics make any overarching conclusions unlikely.

In the networks described before, often controlling every member is unnecessary which makes the control action more ‘expensive’, by which we mean they require more effort, than is necessary. For instance, a predator population in a foodweb may need to be reduced in order to improve a prey population, but other species in the food web may not need be affected. In marketing, an ad agency may want to change the opinion of a certain demographic, but not need to reach every member of the social network. A certain task, sent to a robotic network may need to be performed by only a subset of its members. There are many control goals that can be conceived of for complex networks where the desired final state should only be prescribed for some of the members of the network but not for all of them, which we call target control.

We will show in the following sections that if target control is applicable to a dynamic network, the control energy, or effort, decreases exponentially. We first provide a review of the minimum energy control problem applied to a linear system with the addition of the concept of targeted states. Next, the exponential scaling of the control energy is derived and demonstrated for a moderately sized network (larger examples are contained in the main text for a number of model and real networks). Third, the energy scaling is shown to apply for a control input that is optimal with respect to a more general quadratic cost function (as opposed to the minimum energy formulation introduced in section S2). A comparison between the maximum energy and the average energy for control actions in the p-dimensional output space is then considered. Finally, we provide a referenced table for all the real networks we analyze both here and in the main text.

Supplementary Note 2. Minimum energy output control

The fixed-end point minimum energy control problem is well-known in the optimal control field, especially for a system described by linear dynamics,

$$\begin{aligned}\dot{\mathbf{x}}(t) &= A\mathbf{x}(t) + B\mathbf{u}(t)\\ \mathbf{y}(t) &= C\mathbf{x}(t).\end{aligned} \tag{1}$$

What is less well known is the solution of the minimum energy control problem when the final condition is only prescribed to some subset of the states. We introduce the minimum energy target control problem for networks where the word *target* refers to those nodes with a prescribed final condition. The problem is as follows:

$$\begin{aligned}\min_{\mathbf{u}(t)} \quad J = \frac{1}{2}\int_{t_0}^{t_f} \mathbf{u}^T(t)\mathbf{u}(t)dt&\\ \dot{\mathbf{x}}(t) &= A\mathbf{x}(t) + B\mathbf{u}(t)\\ \mathbf{y}(t) &= C\mathbf{x}(t)\\ \mathbf{x}(t_0) &= \mathbf{x}_0, \quad \mathbf{y}(t_f) = \mathbf{y}_f\end{aligned} \tag{2}$$

The matrix $A \in \mathbb{R}^{n\times n}$ is the adjacency matrix that describes the topology, or inter-connectedness, of the n nodes, or states. The matrix $B \in \mathbb{R}^{n\times m}$ is the control input matrix that describes how the m control inputs are distributed to the nodes. The matrix $C \in \mathbb{R}^{p\times n}$ is the output matrix that relates how each output is a linear combination of the states. For the target control of complex networks formulation, we assume that B (C) has columns (rows) that are all versors, i.e., each control input, $u_i(t)$, $i = 1,\ldots,m$, is directed towards a single node and each output, $y_j(t)$, $j = 1,\ldots,p$, is the state of a single node (see Fig. 1**a** from the main manuscript for a graphical description). The dynamical equation of an arbitrary node i is,

$$\dot{x}_i = \sum_{j=1}^{n} a_{ij}x_j + \sum_{k=1}^{m} b_{ik}u_k \tag{3}$$

where if there exists at least one coefficient $b_{ik} \neq 0$ then node i is what we refer to as an *input node*. We will assume that the system, (A,B,C), is output controllable so that,

$$\operatorname{rank}\left(CB|CAB|\ldots|CA^{n-1}B\right) = p \tag{4}$$

Each output is referred to as a targeted node. The solution of the minimization problem in Eq. (2) is found using Pontryagin's minimum principle [19] and is provided here both as a review and to establish how the targeting aspect of our specific solution is applied. The Hamiltonian equation introduces n costates $\boldsymbol{\nu}(t)$.

$$\mathscr{H}(\mathbf{x}(t),\boldsymbol{\nu}(t),\mathbf{u}(t)) = \frac{1}{2}\mathbf{u}^T(t)\mathbf{u}(t) + \boldsymbol{\nu}^T(t)A\mathbf{x}(t) + \boldsymbol{\nu}^T(t)B\boldsymbol{u}(t) \tag{5}$$

From the Hamiltonian equation, the following dynamical relations can be determined,

$$\begin{aligned}&\text{State Equation:} && \dot{\mathbf{x}}(t) = \frac{\partial\mathscr{H}}{\partial\boldsymbol{\nu}} = A\mathbf{x}(t) + B\mathbf{u}(t)\\ &\text{Costate Equation:} && \dot{\boldsymbol{\nu}}(t) = -\frac{\partial\mathscr{H}}{\partial\mathbf{x}} = -A^T\mathbf{x}(t)\\ &\text{Stationary Equation:} && \mathbf{0} = \frac{\partial\mathscr{H}}{\partial\mathbf{u}} = \mathbf{u}(t) + B^T\boldsymbol{\nu}.\end{aligned} \tag{6}$$

The stationary equation is used to determine the optimal control input.

$$\mathbf{u}^*(t) = -B^T\boldsymbol{\nu} \tag{7}$$

The time evolution of the costates can be determined in a straightforward manner with a final condition of the form, $\boldsymbol{\nu}(t_f) = C^T\hat{\boldsymbol{\nu}}_f$, where $\hat{\boldsymbol{\nu}}_f \in \mathbb{R}^p$ as there are only p final conditions prescribed for the network.

$$\boldsymbol{\nu}(t) = e^{A^T(t_f-t)}C^T\hat{\boldsymbol{\nu}}_f \tag{8}$$

With the optimal control input known, the time evolution of the states can also be determined,

$$\mathbf{x}(t) = e^{A(t-t_0)}\mathbf{x}_0 - \int_{t_0}^{t_f} e^{A(t-\tau)} B B^T e^{A^T(t_f-\tau)} d\tau C^T \hat{\boldsymbol{\nu}}_f \tag{9}$$

The prescribed final condition for the targeted nodes is applied to determine the final, constant vector $\hat{\boldsymbol{\nu}}_f$.

$$\mathbf{y}_f = Ce^{A(t_f-t_0)}\mathbf{x}_0 - CWC^T \hat{\boldsymbol{\nu}}_f \quad \Rightarrow \quad \hat{\boldsymbol{\xi}}_f = -\left(CWC^T\right)^{-1}\left(\mathbf{y}_f - Ce^{A(t_f-t_0)}\mathbf{x}_0\right) \tag{10}$$

The symmetric, positive semi-definite matrix $W = \int_{t_0}^{t_f} e^{A(t_f-\tau)} B B^T e^{A^T(t_f-\tau)} d\tau$ is the controllability Gramian. If the system (A, B, C) is output controllable, then W is positive definite. When C has p rows (versors), the matrix $W_p = CWC^T$, is the output controllability Gramian, and is a $p \times p$ principal submatrix of W.

Supplementary Note 3. Scaling of μ_1

Figures 2, 3, and 4 of the main text provide numerical evidence that the energy required for a control action decreases exponentially as the number of target nodes decreases linearly. In the following derivation, we find that the exponential decay of the energy is a result of a more fundamental property of the output controllability Gramians W_p. Here we show that for a broad class of networks and a random selection of the target nodes the ratio of the smallest eigenvalues of two subsequent principal submatrices of the controllability Gramian W, by which we mean the submatrices W_p and W_{p-1} where W_{p-1} is W_p after removing one additional row-column pair, has a near constant value which we call $\eta_p = \min\{\text{eig}(W_{p-1})\}/\min\{\text{eig}(W_p)\} \approx \textit{constant}$. This is true for a *typical* sequence of random removals of target nodes (here by typical we mean that each node is assigned the same probability of removal and the order of removal is random), while deviations from this behavior are possible for specific removal strategies (see Section S6).

In the main text we have considered the average energy scaling when the cardinality of the target set decreases from j to k, $j > k$. Here, we consider an iterative process as we remove one node at a time from the target set. We say that two target node sets $\mathscr{P}_p$ and $\mathscr{P}_{p+1}$ are adjacent if $\mathscr{P}_{p+1} = \mathscr{P}_p \cup i$ and $i \notin \mathscr{P}_p$.

A symmetric, positive definite matrix $W \in \mathbb{R}^{n \times n}$ has principal submatrices $W_p \in \mathbb{R}^{p \times p}$, $p < n$ where $n - p$ corresponding rows and columns of W have been removed. A principal submatrix, W_p, has diagonal elements which are also diagonal elements of the original matrix W. The eigenvalues of W_p, $\mu_i^{(p)}$, $i = 1, \ldots, p$, are ordered such that,

$$0 < \mu_1^{(p)} \leq \mu_2^{(p)} \leq \ldots \leq \mu_p^{(p)} \tag{11}$$

Consider the case when W_p is W_{p+1} with one additional row-column pair removed, or in terms of the target sets, $\mathscr{P}_p \subset \mathscr{P}_{p+1}$ which are adjacent. From Cauchy's interlacing theorem, the eigenvalues of W_p thread between the eigenvalues of W_{p+1},

$$\mu_1^{(p+1)} \leq \mu_1^{(p)} \leq \mu_2^{(p+1)} \leq \ldots \leq \mu_p^{(p+1)} \leq \mu_p^{(p)} \leq \mu_{p+1}^{(p+1)} \tag{12}$$

The smallest eigenvalue of W_p cannot be smaller than the smallest eigenvalue of W_{p+1}. We perform an iterative process where at each step a row-column pair (without loss of generality here chosen to be the first row and first column) is removed.

$$\begin{aligned} W_{p+1} &= \bar{W}_p + dW_p \\ &= \begin{bmatrix} 0 & \mathbf{0}^T \\ \mathbf{w}_p & W_p \end{bmatrix} + \begin{bmatrix} w_{pp} & \mathbf{w}_p^T \\ \mathbf{0} & O_p \end{bmatrix} \end{aligned} \tag{13}$$

The matrix $\bar{W}_p$ is a $p \times p$ principal submatrix of W_{p+1} with a first row of all zeros and a first column identical to that of W_{p+1}. The matrix dW_p consists of all zeros except for the first row which is identical to the first row of W_{p+1}. The scalar w_{pp} is the leading term in W_{p+1} and $\mathbf{w}_p$ is the first column of W_{p+1}, after removing the entry w_{pp}. Note that the the set of eigenvalues of $\bar{W}_p$ is equal to the set of eigenvalues of W_p with one additional 0 eigenvalue.

The smallest eigenvalue of W_{p+1}, $\mu_1^{(p+1)}$, and the second smallest eigenvalue of $\bar{W}_p$, $\mu_1^{(p)}$ (which is also the smallest eigenvalue of W_p) are used to define the vectors $\mathbf{v}_{p+1}$ and $\bar{\mathbf{v}}_p$,

$$\mathbf{v}_{p+1}^T W_{p+1} = \mathbf{v}_{p+1}^T \mu_1^{(p+1)}, \quad \bar{W}_p \bar{\mathbf{v}}_p = \mu_1^{(p)} \bar{\mathbf{v}}_p \tag{14}$$

Pre- and post-multiplying Eq. (13) by $\mathbf{v}_{p+1}^T$ and $\bar{\mathbf{v}}_p$, respectively, will provide a relation between the smallest eigenvalues of W_{p+1} and W_p.

$$\begin{aligned} \mathbf{v}_{p+1}^T W_{p+1} \bar{\mathbf{v}}_p &= \mathbf{v}_{p+1}^T \bar{W}_p \bar{\mathbf{v}}_p + \mathbf{v}_{p+1}^T dW_p \bar{\mathbf{v}}_p^T \\ \mu_1^{(p+1)} \mathbf{v}_{p+1}^T \bar{\mathbf{v}}_p &= \mu_1^{(p)} \mathbf{v}_{p+1}^T \bar{\mathbf{v}}_p + \mathbf{v}_{p+1}^T W_{p+1} W_{p+1}^{-1} dW_p \bar{\mathbf{v}}_p \\ \mu_1^{(p+1)} &= \mu_1^{(p)} + \mu_1^{(p+1)} \frac{\mathbf{v}_{p+1}^T W_{p+1}^{-1} dW_p \bar{\mathbf{v}}_p}{\mathbf{v}_{p+1}^T \bar{\mathbf{v}}_p} \end{aligned} \tag{15}$$

The matrix product $W_{p+1} dW_p$ is a matrix of all zeros except for the leading term which is one. Thus, the product $\mathbf{v}_{p+1}^T W_{p+1}^{-1} dW_p \bar{\mathbf{v}}_p = [\mathbf{v}_{p+1}]_1 [\bar{\mathbf{v}}_p]_1$ where the notation $[\mathbf{v}]_1$ denotes the first value of a vector. The relation between successive smallest eigenvalues can be written explicitly,

$$\mu_1^{(p)} = \mu_1^{(p+1)}\left(1 - \frac{[\mathbf{v}_{p+1}]_1[\bar{\mathbf{v}}]_1}{\mathbf{v}_{p+1}^T\bar{\mathbf{v}}_p}\right) = \mu_1^{(p+1)}\eta_p \tag{16}$$

We use the definition of the 'worst-case' energy, $E_{\max}^{(p)} = \mu_1^{(p)}$ to rewrite Eq. (16) in terms of energy,

$$E_{\max}^{(p+1)} = E_{\max}^{(p)}\eta_p \quad \Rightarrow \quad \frac{E_{\max}^{(p+1)}}{E_{\max}^{(p)}} = \eta_p \geq 1 \quad \Rightarrow \quad \log E_{\max}^{(p+1)} - \log E_{\max}^{(p)} = \log \eta_p \geq 0 \tag{17}$$

The last of Eq. (17) can be written in terms of any two target sets of size k and j, $k < j$ and $\mathscr{P}_k \subset \mathscr{P}_j$,

$$\log E_{\max}^{(j)} - \log E_{\max}^{(k)} = \sum_{i=k}^{j-1} \log \eta_i \tag{18}$$

We define $\bar{\eta}_{(k\to j)}$, which depends only on the two sets of target nodes $\mathscr{P}_k$ and $\mathscr{P}_j$, as,

$$\log\left(\bar{\eta}_{(k\to j)}^{j-k}\right) = (j-k)\log\bar{\eta}_{(k\to j)} = \sum_{i=k}^{j-1}\log\eta_i \tag{19}$$

In general, there are $\frac{n!}{j!(n-j)!}\frac{j!}{k!(j-k)!} = \frac{n!}{k!(n-j)!(j-k)!}$ possible choices of the sets $\mathscr{P}_k \subset \mathscr{P}_j$ from the n nodes in the network. In the main text, we focus on the specific case when $k = n/10$ and $j = n$ which we use to approximate η,

$$\log E_{\max}^{(n)} - \log E_{\max}^{(n/10)} = (n - \frac{n}{10})\log\bar{\eta}_{(\frac{n}{10}\to n)} \tag{20}$$

Note that for this specific choice of j and k, there are $\frac{n!}{\frac{n}{10}!(n-\frac{n}{10})!}$ choices of end point target sets, or in other words, values of $\log\bar{\eta}_{(\frac{n}{10}\to n)}$. We define η by computing the average of $\log\bar{\eta}_{(\frac{n}{10}\to n)}$,

$$\eta \equiv n\left\langle \log\bar{\eta}_{(\frac{n}{10}\to n)}\right\rangle, \tag{21}$$

where $\langle\cdot\rangle$ is the mean over all possible values. We show in the main text through both model and real network examples that η provides an approximation for $E_{\max}^{(p)}$ such that $\frac{n}{10} \leq p \leq n$, so that we can rewrite Eq. (20) as,

$$\begin{aligned}\left\langle \log E_{\max}^{(p)}\right\rangle &= \left\langle \log E_{\max}^{(n/10)}\right\rangle + \frac{p - n/10}{n}\eta \\ &= \frac{p}{n}\eta + \left(\left\langle \log E_{\max}^{(n/10)}\right\rangle - \frac{1}{10}\eta\right) \\ \left\langle \log E_{\max}^{(p)}\right\rangle &\sim \frac{p}{n}\eta\end{aligned} \tag{22}$$

In Figs. 2, 3 and 4 of the main text, the linear model in the last of Eq. (22) is shown to provide a good approximation of $\log E_{\max}^{(p)}$. In Fig. 1, from Eqs. (18) and (19) we set $k = p_{\min} = n/10$, or 10% of the nodes in the network, and let $j = p_{\max}$ increase from 30% to 90%, to show how the standard deviation of $\log\bar{\eta}_{(p_{\min}\to p_{\max})}$ (that is of the $\log E_{\max}^{(p_{\max})}$, see Eq. (18)) decreases as we increase the cardinality of the target sets. As we consider more values of η_i corresponding to larger values of $p_{\max}$, the peak of the PDF grows, meaning the variation of values of $\log E_{\max}^{(p_{\max})}$ decreases. As we demonstrate the variation of $\log\bar{\eta}_{p_{\min}\to p_{\max}}$ becomes small when $p_{\max} - p_{\min}$ increases, we can rewrite Eq. (19) as approximately

$$(p_{\max} - p_{\min})\log\bar{\eta}_{p_{\min}\to p_{\max}} \approx (p_{\max} - p_{\min})\langle\log\eta_i\rangle \tag{23}$$

where $i = p_{\min}, \dots, p_{\max}$. It is seen through experiments that $\langle\log\eta_i\rangle$ is independent of the target set size (a generic example is shown in Fig. 2) and can be computed for a given network. We stress that while we have not proven η_i is independent of the target node set cardinality i, we have provided ample numerical evidence through the exponential scaling as seen in Figs. 2, 3, and 4 in the main text that η_i is invariant. The network parameter η can be approximated simply as,

$$\eta \approx n\langle\eta_i\rangle \tag{24}$$

as η_i can be approximated as being constant. In Fig. 2 we show an example of when η_i is approximately constant and how η, the energy scaling value, can be closely approximated by assuming η_i is constant. The decrease of the standard deviation for each distribution is shown with respect to $p_{\max}$ in the inset.

Supplementary Note 4. The application of the algebraic Riccati equation to finite horizon LQ problems

In this section, we derive a closed form solution of the linear quadratic optimal control problem in a Gramian-like form similar to the well-known solution of the minimum energy optimal control problem. Note that the classical solution to the LQ optimal control problem looks for a control input that is a function of only the states by computing approximate solutions to the differential Riccati equation [19]. The difference between the classical solution and the solution provided here is that there is an 'open-loop' portion of the control input, i.e., not dependent on the current state. The closed-form solution is available after a similarity transformation as explained below. The LQ optimal control problem is laid out below,

$$\begin{aligned} \min_{\mathbf{u}(t)} \quad & J = \frac{1}{2}\int_{t_0}^{t_f} \left[\mathbf{x}^T(t)Q\mathbf{x}(t) + 2\mathbf{x}^T(t)M\mathbf{u}(t) + \mathbf{u}^T(t)R\mathbf{u}(t)\right] dt \\ & \dot{\mathbf{x}}(t) = A\mathbf{x}(t) + B\mathbf{u}(t) \\ & \mathbf{y}(t) = C\mathbf{x}(t) \\ & \mathbf{x}(t_0) = \mathbf{x}_0, \quad \mathbf{y}(t_\mathrm{f}) = \mathbf{y}_\mathrm{f} \end{aligned} \tag{25}$$

The problem above considers systems with n states $x_i(t)$, $i = 1,\ldots,n$, m control inputs $u_j(t)$, $j = 1,\ldots,m$, and p outputs $y_k(t)$, $k = 1,\ldots,p$. We assign the same properties to B and C as we did previously, that the columns (rows) of B (C) are all linearly independent versors. The state weight matrix, $Q \in \mathbb{R}^{n\times n}$, must be real, symmetric and positive semi-definite. The mixed weight matrix, $M \in \mathbb{R}^{n\times m}$, must be real. The control input weight matrix, $R \in \mathbb{R}^{m\times m}$, must be real, symmetric, and positive definite. The time horizon, $\delta t = t_\mathrm{f} - t_0$, $t_0 < t_\mathrm{f}$, dictates the time desired to move the system from an initial condition, defined for every state, to the final condition defined for *only* the targets. We will use Pontryagin's minimum principle [19] to calculate the optimal control input, $\mathbf{u}_\mathrm{c}^*(t)$. The Hamiltonian equation is defined which introduces n time-varying costates $\boldsymbol{\nu}(t)$,

$$\mathscr{H}(\mathbf{x}(t),\boldsymbol{\nu}(t),\mathbf{u}(t)) = \frac{1}{2}\mathbf{x}^T(t)Q\mathbf{x}(t) + \mathbf{x}^T(t)M\mathbf{u}(t) + \frac{1}{2}\boldsymbol{u}^T(t)R\mathbf{u}(t) + \boldsymbol{\nu}^T(t)A\mathbf{x}(t) + \boldsymbol{\nu}(t)B\mathbf{u}(t) \tag{26}$$

The method defines three equations that, if satisfied, guarantees an optimal solution with respect to Eq. (25),

$$\begin{aligned} &\text{State Equation:} && \dot{\mathbf{x}}(t) = \frac{\partial \mathscr{H}}{\partial \boldsymbol{\nu}(t)} = A\mathbf{x}(t) + B\mathbf{u}(t) \\ &\text{Costate Equation:} && \dot{\boldsymbol{\nu}}(t) = -\frac{\partial \mathscr{H}}{\partial \mathbf{x}(t)} = -Q\mathbf{x}(t) - M\mathbf{u}(t) - A^T\boldsymbol{\nu}(t) \\ &\text{Stationary Equation:} && \mathbf{0} = \frac{\partial \mathscr{H}}{\partial \mathbf{u}(t)} = M^T\mathbf{x}(t) + R\mathbf{u}(t) + B^T\boldsymbol{\nu}(t) \end{aligned} \tag{27}$$

The stationary equation provides the optimal control input, $\boldsymbol{u}^*(t)$,

$$\boldsymbol{u}_\mathrm{c}^*(t) = -R^{-1}\left(M^T\boldsymbol{x}(t) + B^T\boldsymbol{\nu}(t)\right). \tag{28}$$

What remains is to solve the dynamical system defined by the state and costate equations in Eq. (27). First, Eq. (28) is applied to the state and costate equations to make the system homogeneous,

$$\begin{aligned} \dot{\mathbf{x}}(t) &= A\mathbf{x}(t) + B\left(-R^{-1}\left(M^T\mathbf{x}(t) + B^T\boldsymbol{\nu}(t)\right)\right) \\ &= \left(A - BR^{-1}M^T\right)\mathbf{x}(t) - BR^{-1}B^T\boldsymbol{\nu}(t), \end{aligned} \tag{29}$$

$$\begin{aligned} \dot{\boldsymbol{\nu}}(t) &= -Q\mathbf{x}(t) - M\left(-R^{-1}\left(M^T\mathbf{x}(t) + B^T\boldsymbol{\nu}(t)\right)\right) - A^T\boldsymbol{\nu}(t) \\ &= \left(MR^{-1}M^T - Q\right)\mathbf{x}(t) + \left(MR^{-1}B^T - A^T\right)\boldsymbol{\nu}(t). \end{aligned} \tag{30}$$

The homogeneous Hamiltonian system is the following linear equation,

$$\begin{bmatrix} \dot{\mathbf{x}}(t) \\ \dot{\boldsymbol{\nu}}(t) \end{bmatrix} = \begin{bmatrix} A - BR^{-1}M^T & -BR^{-1}B^T \\ MR^{-1}M^T - Q & -A^T + MR^{-1}B^T \end{bmatrix} \begin{bmatrix} \mathbf{x}(t) \\ \boldsymbol{\nu}(t) \end{bmatrix} \tag{31}$$

Note that dynamical equations for the states and costates are coupled but an initial condition is imposed on the state variables, while a final condition is imposed on the costate variables. Because of the coupled nature of the problem, we cannot compute the state and costate trajectories individually. We use the relation between the costate and the state, $\boldsymbol{\nu}(t) = S\mathbf{x}(t) + \boldsymbol{\xi}(t)$, where S is restricted to be symmetric, to define a similarity transformation for the matrix in Eq. (31). We then obtain,

$$\begin{bmatrix} \mathbf{x}(t) \\ \boldsymbol{\nu}(t) \end{bmatrix} = \begin{bmatrix} I & O \\ S & I \end{bmatrix} \begin{bmatrix} \mathbf{x}(t) \\ \boldsymbol{\xi}(t) \end{bmatrix} \tag{32}$$

The matrix in Eq. (32) has the following inversion property:

$$\begin{bmatrix} I & O \\ S & I \end{bmatrix}^{-1} = \begin{bmatrix} I & O \\ -S & I \end{bmatrix} \tag{33}$$

We use the relation in Eq. (32) to rewrite Eq. (31) so that $\dot{\boldsymbol{\xi}}(t)$ is decoupled from the states, $\mathbf{x}(t)$:

$$\begin{aligned} \begin{bmatrix} \dot{\mathbf{x}}(t) \\ \dot{\boldsymbol{\xi}}(t) \end{bmatrix} &= \begin{bmatrix} I & O \\ -S & I \end{bmatrix} \begin{bmatrix} A - BR^{-1}M^T & -BR^{-1}B^T \\ MR^{-1}M^T - Q & -A^T + MR^{-1}B^T \end{bmatrix} \begin{bmatrix} I & O \\ S & I \end{bmatrix} \begin{bmatrix} \mathbf{x}(t) \\ \boldsymbol{\xi}(t) \end{bmatrix} \\ &= \begin{bmatrix} \tilde{A} & \tilde{B} \\ \tilde{Q} & -\tilde{A}^T \end{bmatrix} \begin{bmatrix} \mathbf{x}(t) \\ \boldsymbol{\xi}(t) \end{bmatrix} \end{aligned} \tag{34}$$

The matrix $\tilde{A}$ is defined as the augmented adjacency matrix because of its similar role in the state costate system,

$$\tilde{A} = A - BR^{-1}M^T - BR^{-1}B^T S. \tag{35}$$

The matrix $\tilde{B}$ acts as a control input matrix where $\boldsymbol{\xi}(t)$ acts as a pseudo control input,

$$\tilde{B} = -BR^{-1}B^T. \tag{36}$$

Our desire is to define S such that $\tilde{Q}$ is a zero matrix and so that the states, $\mathbf{x}(t)$, are decoupled from $\boldsymbol{\xi}(t)$,

$$\tilde{Q} = S\left(A - BR^{-1}M^T\right) + \left(A^T - MR^{-1}B^T\right)S - SBR^{-1}B^T S + Q - MR^{-1}M^T = O_n. \tag{37}$$

Note that Eq. (37) is in standard algebraic Riccati equation form with respect to S. There are a number of routines which provide a solution to Eq. (37), and we can, with confidence, rewrite Eq. (34) such that $\boldsymbol{\xi}(t)$ is decoupled from $\mathbf{x}(t)$,

$$\begin{bmatrix} \dot{\mathbf{x}}(t) \\ \dot{\boldsymbol{\xi}}(t) \end{bmatrix} = \begin{bmatrix} \tilde{A} & \tilde{B} \\ O_n & -\tilde{A}^T \end{bmatrix} \begin{bmatrix} \mathbf{x}(t) \\ \boldsymbol{\xi}(t) \end{bmatrix}. \tag{38}$$

The solution for the alternate costate, $\boldsymbol{\xi}(t)$, is written in terms of a final condition, $\boldsymbol{\xi}(t_f) = \boldsymbol{\xi}_f$.

$$\boldsymbol{\xi}(t) = e^{\tilde{A}^T(t_\mathrm{f}-t)}\boldsymbol{\xi}_\mathrm{f} \tag{39}$$

As there are p final outputs, we rewrite the final costate condition, $\boldsymbol{\xi}_\mathrm{f} = C^T\hat{\boldsymbol{\xi}}_\mathrm{f}$. With the equation for the alternate costate trajectory in Eq. (39), the time evolution of the states can be computed.

$$\begin{aligned} \mathbf{x}(t) &= e^{\tilde{A}(t-t_0)}\mathbf{x}_0 + \int_{t_0}^{t} e^{\tilde{A}(t-\tau)}\tilde{B}\boldsymbol{\xi}(\tau)d\tau \\ &= e^{\tilde{A}(t-t_0)}\mathbf{x}_0 + \int_{t_0}^{t} e^{\tilde{A}(t-\tau)}\tilde{B}e^{\tilde{A}^T(t_\mathrm{f}-\tau)}d\tau C^T\hat{\boldsymbol{\xi}}_\mathrm{f} \end{aligned} \tag{40}$$

All that remains is to apply the final output to the time evolution of the targets to define the vector $\hat{\boldsymbol{\xi}}(t)$.

$$\mathbf{y}_\mathrm{f} = Ce^{\tilde{A}(t_\mathrm{f}-t_0)}\mathbf{x}_0 + C\tilde{W}C^T\hat{\boldsymbol{\xi}}_\mathrm{f} \quad \Rightarrow \quad \hat{\boldsymbol{\xi}}_\mathrm{f} = \left(C\tilde{W}C^T\right)^{-1}\left(\mathbf{y}_\mathrm{f} - Ce^{\tilde{A}(t_\mathrm{f}-t_0)}\mathbf{x}_0\right) \tag{41}$$

The matrix $\tilde{W} = \int_{t_0}^{t_\mathrm{f}} e^{\tilde{A}(t_\mathrm{f}-\tau)}\tilde{B}e^{\tilde{A}^T(t_\mathrm{f}-\tau)}d\tau$ is defined as the generalized controllability Gramian. We also can define the control maneuver as $\tilde{\boldsymbol{\beta}} = \mathbf{y}_\mathrm{f} - Ce^{\tilde{A}(t_\mathrm{f}-t_0)}\mathbf{x}_0$. The optimal control input is written in terms of the state and alternate costate solutions,

$$
\begin{aligned}
\mathbf{u}_{\mathrm{c}}^{*}(t) &= -R^{-1}M^{T}\mathbf{x}(t) - R^{-1}B^{T}\left(S\mathbf{x}(t) + \boldsymbol{\xi}(t)\right) \\
&= \left(-R^{-1}M^{T} - R^{-1}B^{T}S\right)\mathbf{x}(t) - R^{-1}B^{T}\boldsymbol{\xi}(t) \\
&= L\mathbf{x}(t) - R^{-1}B^{T}e^{\tilde{A}^{T}(t_{\mathrm{f}}-\tau)}C^{T}\left(C\tilde{W}C^{T}\right)^{-1}\tilde{\boldsymbol{\beta}}.
\end{aligned} \tag{42}
$$

The optimal control input is the sum of a linear combination of the states where $L = -R^{-1}(M^{T} + B^{T}S)$ and the open-loop portion which, through the final costate condition in Eq. (41), is a function of the initial condition of the states and the final condition on the outputs, or more specifically, the targets.

The energy of the control action is defined as the cumulative effort of the control signal, $\mathbf{u}_{\mathrm{c}}^{*}(t)$.

$$
\begin{aligned}
E_{\mathrm{c}}^{(p)} &= \int_{t_0}^{t_{\mathrm{f}}} \mathbf{u}_{\mathrm{c}}^{*T}(t)\mathbf{u}_{\mathrm{c}}^{*}(t)dt \\
&= \int_{t_0}^{t_{\mathrm{f}}} \Big[\mathbf{x}^{T}(t)L^{T}L\mathbf{x}(t) - 2\mathbf{x}^{T}(t)L^{T}R^{-1}B^{T}e^{\tilde{A}^{T}(t_{\mathrm{f}}-t)}C^{T}\left(C\tilde{W}C^{T}\right)^{-1}\tilde{\boldsymbol{\beta}} \\
&\quad + \tilde{\boldsymbol{\beta}}^{T}\left(C\tilde{W}C^{T}\right)^{-1}Ce^{\tilde{A}(t_{\mathrm{f}}-t)}BR^{-2}B^{T}e^{\tilde{A}^{T}(t_{\mathrm{f}}-t)}C^{T}\left(C\tilde{W}C^{T}\right)^{-1}\tilde{\boldsymbol{\beta}}\Big]dt
\end{aligned} \tag{43}
$$

When the controllability Gramian is poorly conditioned, which is often the case in the control of large complex networks (as shown in Fig. 8 of the main text), it is possible to approximate the energy as the integral of only the final term in Eq. (43).

$$
E_{\mathrm{c}}^{(p)} \approx \tilde{\boldsymbol{\beta}}^{T}\left(C\tilde{W}C^{T}\right)^{-1}C\int_{t_0}^{t_{\mathrm{f}}} e^{\tilde{A}(t_{\mathrm{f}}-t)}BR^{-2}B^{T}e^{\tilde{A}^{T}(t_{\mathrm{f}}-t)}dtC^{T}\left(C\tilde{W}C^{T}\right)^{-1}\tilde{\boldsymbol{\beta}} \tag{44}
$$

If $\tilde{B} = B\hat{R}^{-1}B^{T} = B\hat{R}^{-2}B^{T}$, which would be true if $R = I$ for instance, then the integral in Eq. (44) is the generalized controllability Gramian and the energy is of a quadratic form,

$$
\begin{aligned}
E_{\mathrm{c}}^{(p)} &\approx \tilde{\boldsymbol{\beta}}^{T}\left(C\tilde{W}C^{T}\right)^{-1}C\tilde{W}C^{T}\left(C\tilde{W}C^{T}\right)^{-1}\tilde{\boldsymbol{\beta}} \\
&= \tilde{\boldsymbol{\beta}}^{T}\left(C\tilde{W}C^{T}\right)^{-1}\tilde{\boldsymbol{\beta}}.
\end{aligned} \tag{45}
$$

The vector $\tilde{\boldsymbol{\beta}}$ which we call the *control maneuver*, has information about the initial and final condition of the system. We see from Eq. (43) that the optimal control input is actually the sum of two distinct components, $\mathbf{u}_{\mathrm{c}1}^{*}$ and $\mathbf{u}_{\mathrm{c}2}^{*}$. However our approximation in Eq. (45) only takes into consideration $\mathbf{u}_{\mathrm{c}2}^{*}$. We see that for both model networks in Fig. 4**a** & 4**b** and datasets from the literature (*IEEE 118 bus test grid* [7] and a *Florida food web* [1]) in Fig. 4**c** with various target set sizes, p/n, the open loop control energy $E_{\max}^{(p)} \approx \tilde{\boldsymbol{\beta}}^{T}W_p^{-1}\tilde{\boldsymbol{\beta}}$ well approximates the full control energy $\mathbf{u}^{*}(t) = \mathbf{u}_{\mathrm{c}1}^{*}(t) + \mathbf{u}_{\mathrm{c}2}^{*}(t)$. The solid line has a slope of one in all three panels and each of the points nearly lies upon it. In the paper, we construct our cost function such that there is a weight applied to both the states and the state derivative. The definition of the time derivative of the states from Eq. (1) is used to rewrite the cost function in Eq. (46) in the form of Eq. (25),

$$
\begin{aligned}
J &= \frac{1}{2}\int_{t_0}^{t_{\mathrm{f}}} \left[\dot{\mathbf{x}}^{T}(t)Q_1\dot{\mathbf{x}}(t) + \mathbf{x}^{T}(t)Q_1\mathbf{x}(t) + \mathbf{u}^{T}(t)\hat{R}\mathbf{u}(t)\right]dt \\
&= \frac{1}{2}\int_{t_0}^{t_{\mathrm{f}}} \left[\left(\mathbf{u}^{T}(t)B^{T} + \mathbf{x}^{T}(t)A^{T}\right)Q_1\left(A\mathbf{x}(t) + B\mathbf{u}(t)\right) + \mathbf{x}^{T}(t)Q_2\boldsymbol{x}(t) + \mathbf{u}^{T}(t)\hat{R}\mathbf{u}(t)\right]dt \\
&= \frac{1}{2}\int_{t_0}^{t_{\mathrm{f}}} \left[\mathbf{x}^{T}(t)\left(A^{T}Q_1A + Q_2\right)\mathbf{x}(t) + 2\mathbf{x}^{T}(t)\left(A^{T}Q_1B\right)\mathbf{u}(t) + \mathbf{u}^{T}(t)\left(B^{T}Q_1B + \hat{R}\right)\mathbf{u}(t)\right]dt
\end{aligned} \tag{46}
$$

Comparing Eqs. (46) and (25) provides the relations $Q = A^{T}Q_1A + Q_2$, $M = A^{T}Q_1B$, and $R = B^{T}Q_1B + \hat{R}$. We consider the formulation in Eq. (46) and provide an example in Fig. 3 of how including a weight on the state derivative can substantially change the path of the trajectory. The solid lines correspond to the minimum energy trajectories as they were derived in section S2. The dotted lines correspond to the minimum cost trajectories derived in this section where a weight has been added to the derive of x_2 and x_3. We see that the state trajectories of nodes 2 and 3 have become straighter (diminishing the length of the trajectory) while the input node, node 1, has now experienced a sharp increase at the beginning of its evolution and sharp decrease at the end of its trajectory. More careful tuning can allow for many different shaped trajectories.

Supplementary Note 5. Average Energy vs Worst-Case Energy

Here we will show that the order of the worst-case energy dominates the energy needed to reach any coordinate in state space. Consider a system with p targets, with initial condition at the origin and final output located on the p-dimension unit hyper-sphere (this hyper-sphere is defined in the p-dimensional subspace of phase space corresponding to the p target states). The average energy required to reach a location on the p-dimensional unit hyper-sphere is determined from,

$$E_{\rm av}^{(p)} = \frac{1}{p}\sum_{i=1}^{p} E^{(p)} = \frac{1}{p}\sum_{i=1}^{p} \boldsymbol{v}_p^T W_p^{-1} \boldsymbol{v}_p = \frac{1}{p}\sum_{i=1}^{p} \frac{1}{\mu_i^{(p)}} \tag{47}$$

where the vector $\boldsymbol{v}_p \in \mathbb{R}^p$ and $||\boldsymbol{v}_p|| = 1$. Note that the vector $\boldsymbol{\beta}$, which we call the control maneuver, can be written as a weighted linear combination of the normalized eigenvectors of W_p^{-1},

$$\boldsymbol{\beta} = \sum_{i=1}^{p} a_i \boldsymbol{v}_i \tag{48}$$

We use the summation in Eq. (48) to write Eq. (47) in terms of the eigenvectors of W_p^{-1}.

$$\boldsymbol{\beta}^T W_p^{-1} \boldsymbol{\beta} = \sum_{i=1}^{p}\sum_{j=1}^{p} a_i a_j \boldsymbol{v}_i^T W_p^{-1} \boldsymbol{v}_j = \sum_{i=1}^{p} a_i^2 \frac{1}{\mu_i^{(p)}} \sim E_{\rm av}^{(p)} \tag{49}$$

For scale-free networks, we have seen the largest eigenvalue of W_p^{-1}, $\frac{1}{\mu_1}$, is far larger than any of the other eigenvalues and thus the summation in Eq. (49) is dominated by $\frac{1}{\mu_1^{(p)}}$.

A demonstration of the role $1/\mu_1^{(p)}$ has in the average energy is displayed in Fig. 5. The maximum energy is calculated in the usual way, $E_{\rm max}^{(p)} = 1/\mu_1^{(p)}$, and the average energy is calculated as in Eq. (47). The two curves, for any value of p/n, appear close to each other demonstrating the dominance of $1/\mu_1^{(p)}$ in the summation of the inverse eigenvalues of W_p.

Supplementary Note 6. Target Choice Affects Energy

So far we have investigated how the maximum energy is exponentially dependent on the target set size. The relation $\log E_{\max}^{(p)} = \frac{p}{n}\eta + constant$, where η is network specific, lets us compute the maximum energy if η is known. To estimate η for a network, we have typically computed many values of η_p, $p = 1, \ldots, n$ where the p nodes in the target set are sampled randomly and the mean of η_p is taken to be $\bar{\eta}$. We now consider the case if the target nodes are not chosen randomly, but instead, chosen with respect to the in-degree or out-degree of the individual nodes. Figure 6 further investigates the dependece of $E_{\max}^{(p)}$ on different selection strategies for the target nodes. Namely, the target nodes were chosen in order of ascending in-degree (AI), descending in-degree (DI), ascending out-degre (AO), and descending out-degree (DO). As can be seen, when these strategies are considered, $E_{\max}^{(p)}$ decreases in a way that strongly depends on the particular strategy applied and substantially differs from network to network, i.e., it is network specific.

References

[1] http://vlado.fmf.uni-lj.si/pub/networks/data/.

[2] Martinez, N. D. Artifacts or attributes? effects of resolution on the little rock lake food web. *Ecological Monographs* **61**, 367–392 (1991).

[3] Milo, R. *et al.* Superfamilies of evolved and designed networks. *Science* **303**, 1538–1542 (2004).

[4] Lafferty, K. D., Hechinger, R. F., Shaw, J. C., Whitney, K. & Kuris, A. M. Food webs and parasites in a salt marsh ecosystem. *Disease ecology: community structure and pathogen dynamics* 119–134 (2006).

[5] Hall, S. & Raffaelli, D. Food-web patterns: lessons from a species-rich web. *The Journal of Animal Ecology* **60**, 823–841 (1991).

[6] http://research.mssm.edu/maayan/datasets/qualitative_networks.shtml.

[7] https://www.ee.washington.edu/research/pstca/pf118/pg_tca118bus.htm.

[8] Menck, P. J., Heitzig, J., Kurths, J. & Schellnhuber, H. J. How dead ends undermine power grid stability. *Nat. Commun.* **5** (2014).

[9] Colizza, V., Pastor-Satorras, R. & Vespignani, A. Reaction–diffusion processes and metapopulation models in heterogeneous networks. *Nature Physics* **3**, 276–282 (2007).

[10] Jeong, H., Tombor, B., Albert, R., Oltvai, Z. N. & Barabási, A.-L. The large-scale organization of metabolic networks. *Nature* **407**, 651–654 (2000).

[11] Yu, H. *et al.* Next-generation sequencing to generate interactome datasets. *Nature methods* **8**, 478–480 (2011).

[12] http://interactome.dfci.harvard.edu/S_cerevisiae/index.php.

[13] Yu, H. *et al.* High-quality binary protein interaction map of the yeast interactome network. *Science* **322**, 104–110 (2008).

[14] Guimera, R., Danon, L., Diaz-Guilera, A., Giralt, F. & Arenas, A. Self-similar community structure in a network of human interactions. *Physical review E* **68**, 065103 (2003).

[15] Opsahl, T. Triadic closure in two-mode networks: Redefining the global and local clustering coefficients. *Social Networks* **35**, 159–167 (2013).

[16] Gleiser, P. M. & Danon, L. Community structure in jazz. *Adv. Complex Syst.* **6** (2003).

[17] Freeman, L. C., Webster, C. M. & Kirke, D. M. Exploring social structure using dynamic three-dimensional color images. *Social Networks* **20**, 109–118 (1998).

[18] Opsahl, T. & Panzarasa, P. Clustering in weighted networks. *Social networks* **31**, 155–163 (2009).

[19] Kirk, D. E. *Optimal control theory: an introduction* (Courier Corporation, 2012).
